# Gossip Versus Punishment: The Efficiency of Reputation to Promote and Maintain Cooperation

**DOI:** 10.1038/srep23919

**Published:** 2016-04-04

**Authors:** Junhui Wu, Daniel Balliet, Paul A. M. Van Lange

**Affiliations:** 1 Vrije Universiteit Amsterdam, Department of Experimental and Applied Psychology, Van der Boechorststraat 1, 1081 BT Amsterdam, the Netherlands

## Abstract

Prior theory suggests that reputation spreading (e.g., gossip) and punishment are two key mechanisms to promote cooperation in groups, but no behavioral research has yet examined their relative effectiveness and efficiency in promoting and maintaining cooperation. To examine these issues, we observed participants interacting in a four-round public goods game (PGG) with or without gossip and punishment options, and a subsequent two-round trust game (TG). We manipulated gossip as the option to send notes about other group members to these members’ future partners, and punishment as the option to assign deduction points to reduce other group members’ outcomes with a fee-to-fine ratio of 1:3. Findings revealed that in the four-round PGG, the option to gossip increased both cooperation and individual earnings, whereas the option to punish had no overall effect on cooperation (but a positive effect on cooperation in the last two rounds of the PGG) and significantly decreased individual earnings. Importantly, the initial option to gossip made people more trusting and trustworthy in the subsequent TG when gossip was no longer possible, compared to the no-gossip condition. Thus, we provide some initial evidence that gossip may be more effective and efficient than punishment to promote *and* maintain cooperation.

Cooperation is essential for groups, organizations, and societies to achieve and maintain public goods that benefit all group members. However, cooperation to provide public goods usually requires people to overcome the temptation to free ride and take advantage of others’ cooperation. Why do people cooperate despite this temptation to prioritize their own interests? Previous theory suggests that reputation (e.g., gossip) and punishment are two key mechanisms to promote cooperation in groups^1^,^2^,^3^. Yet, little is known about the relative difference between gossip and punishment to promote cooperation and what happens to cooperation rates after these mechanisms are removed in future interactions^4^. We extend previous research by directly comparing gossip and punishment in their relative ability to (1) promote cooperation, (2) enhance individual welfare, and (3) maintain cooperation in future interactions with no such mechanisms.

One of the most widely studied solutions to cooperation problems involves opportunities to punish others’ (selfish) behavior^1^,^2^. Punishment decreases the incentive to free ride and makes cooperation relatively more beneficial for individuals. Indeed, punishment reduces the conflict between personal and collective interests in providing the public good, and thus increases cooperation^1^,^2^. Yet, punishment is costly for individuals and groups, and is not always a feasible solution to promote cooperation^5^. Moreover, although people do punish free riders during controlled experiments when given the chance^2^,^6^, peer punishment can be uncommon in real-life situations in both small-scale and large-scale societies^7^,^8^. Thus, there is a need to generate other effective solutions to promote cooperation in groups.

Importantly, people are prone to gossip about norm violators and free riders, and this gossip facilitates reputation spreading in large groups and social networks^9^,^10^. Gossip is the exchange of positive or negative social information about absent others^10^,^11^,^12^,^13^. Thus, gossip can influence others’ reputation and enable people to select trustworthy partners and avoid cheaters, especially in large groups where directly observing all social interactions is not possible. Both simulation models and empirical studies suggest that reputation monitoring and exchange via gossip serves as a low-cost and efficient mechanism to promote cooperation^11^,^12^,^13^,^14^. Reputation facilitates cooperation through a system of indirect reciprocity, where people with a cooperative reputation have greater chance to receive future indirect benefits from third parties^3^. Indeed, reputation and indirect reciprocity can promote cooperation and facilitate partner selection in social interactions^15^,^16^. People tend to be more cooperative when their reputation is at stake^11^,^13^,^14^, and also condition their own cooperation on others’ reputation through gossip^17^. Therefore, people should be motivated to enhance their reputation by acting more cooperatively when there is potential gossip to their future partner, compared to situations with no gossip opportunities. But how effective are gossip and reputation in promoting cooperation, relative to punishment?

A recent agent-based simulation compared the effects of gossip and punishment on cooperation in a public goods game where the agents decided whether or not to contribute a fixed amount to the public good. The results suggested that both gossip-based partner selection and costly punishment increased cooperation rates, and cooperation was higher when these two were combined^18^. However, to our knowledge, this prediction has not yet been tested in behavioral experiments. We predict that both gossip and punishment should increase cooperation. Moreover, we examine their relative effectiveness and provide an empirical test of the prediction from simulation studies: gossip and punishment combined will promote greater cooperation than gossip or punishment alone^18^.

Despite their similar positive effects on cooperation, gossip and punishment might affect individual welfare differently. Punishment is often costly to the punisher and more severely reduces the outcomes of those being punished, and this leads to its negative effects on both individual and group efficiency^19^. Yet, the inefficiency of punishment has mostly been established in short-term (one-shot) interactions, but there is evidence that punishment can become a relatively more efficient strategy promoting cooperation over long-term interactions^20^. Gossip, however, involves less cost in information exchange without lowering its effectiveness in promoting cooperation^12^. Moreover, people can face decisions about whether to gossip about and/or punish free riders in social interactions. At least initially, people should prefer to gossip about free riders (or other cooperators) with less cost rather than punish free riders with more cost. For this reason, the option to gossip may counter the detrimental effect of punishment on individual welfare. Thus, we predict that participants would (a) gain *less* benefit from social interactions with options to punish, compared to no-punishment situations, (b) gain *more* benefit from social interactions with options to gossip, compared to no-gossip situations, and (c) gain *more* (or *less*) benefit in situations with both options to gossip and punish than in situations with the only option to punish (or gossip).

Several scholars have argued that the tendency to cooperate can be rooted in social preferences, particularly concerns for others’ welfare^21^,^22^,^23^,^24^. Yet, people can also behave cooperatively due to monetary punishment or their concerns about reputation^1^,^25^. Previous theorizing and research have established that punishment is an extrinsic incentive that can undermine one’s intrinsic motivation to cooperate, make people less cooperative and less likely to trust others to voluntarily cooperate when this incentive is removed in future interactions^26^,^27^,^28^,^29^. Thus, punishment may have long-term negative consequences when one encounters future situations without punishment. In contrast, the belief that others can gossip about oneself can motivate people to enhance their reputation by being more cooperative, because (costly) cooperation signals one’s willingness to incur a cost for group welfare and quality as a trustworthy partner^14^,^30^. Importantly, people may also tend to maintain their cooperation once they have built up a good reputation, as previous research suggests that a good reputation is hard to gain but easy to lose^31^. Thus, gossip may have some “sustainable effects” in future interactions with no such mechanism. We attempt to test this possibility by comparing how the initial presence of gossip and punishment during social interactions affect cooperation in subsequent interactions that lack opportunities for gossip and punishment. Accordingly, we predict that (a) the initial option to punish each other would make people less cooperative once they are unable to punish in future interactions, whereas (b) the initial option to gossip about each other would make people more cooperative even when there is no option to gossip in future interactions.

To test our hypotheses, we conducted a real-time interaction experiment with 265 participants recruited from Amazon Mechanical Turk (MTurk). In this computerized interaction paradigm, participants interacted with others online in a four-round public goods game (PGG) with gossip and punishment manipulations, and a subsequent two-round trust game (TG) as an investor and a responder, respectively. We measured participants’ cooperation in the PGG, and their levels of trust and trustworthiness in the TG. The experiment was a 2 (no gossip vs. gossip) × 2 (no punishment vs. punishment) between-subjects design with four conditions: (a) *control* condition with no option to gossip or punish (*n* = 63), (b) *punishment* condition (*n* = 69), (c) *gossip* condition (*n* = 67), and (d) *gossip-and-punishment* condition (*n* = 66). We observed how the levels of cooperation and the efficiency of providing public goods (i.e., individual earnings in the PGG) varied in response to the option to gossip or punish. We also examined behavior in the subsequent TG with no gossip or punishment to test whether the initial option to gossip and/or punish can maintain cooperation. As an initial experiment on gossip and punishment, we consider gossip to involve no financial cost, and punishment with a moderate cost that is most commonly used in prior research (i.e., fee-to-fine ratio of 1:3)^1^.

## Results

### Gossip versus punishment: Effectiveness in promoting cooperation

**Figure 1:**
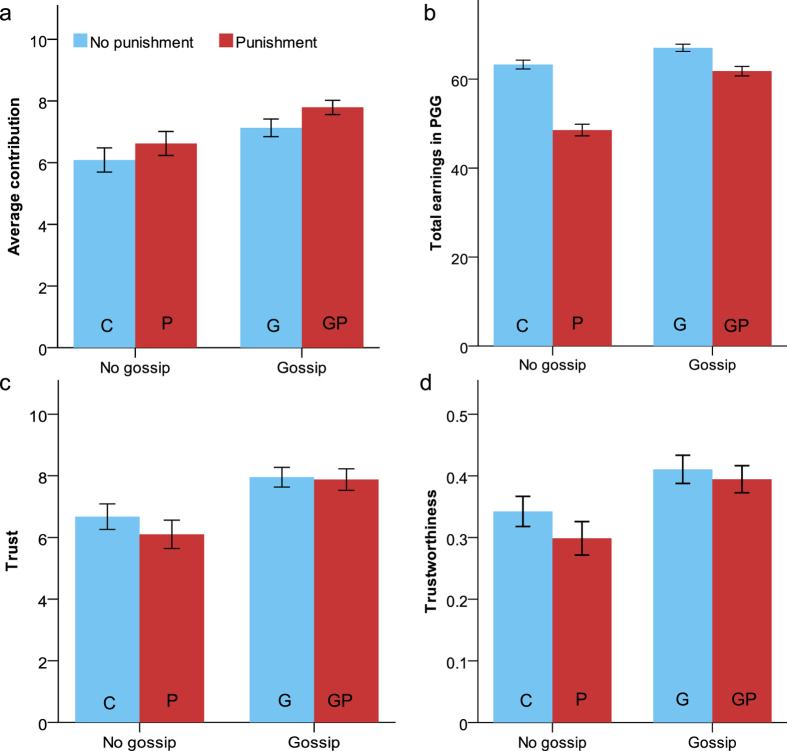
Relative effectiveness and efficiency of gossip and punishment in promoting and maintaining cooperation. (**a**) Average contribution and (**b**) total earnings in the PGG, (**c**) trust, and (**d**) trustworthiness in the TG as a function of gossip and punishment manipulations. C = control condition with no gossip or punishment, P = punishment condition, G = gossip condition, GP = gossip-and-punishment condition. Error bars indicate standard errors of the mean.

The average contributions across the four-round PGG in each condition are presented in Fig. 1a. We used linear mixed model (LMM) analysis (SPSS v. 23) to fit the data with contribution as the dependent variable, and gossip and punishment as between-subjects factors. Round number as a repeated measure was specified with an unstructured covariance matrix. The variance across sessions was modeled with a random intercept^32^. We found a marginally significant main effect of gossip, *F*(1, 15.454) = 4.460, *p* = 0.051, with more contribution in each round when participants could gossip (*M* = 7.444, *SE* = 0.357), compared to unable to gossip (*M* = 6.378, *SE* = 0.357). The main effect of punishment was not significant, *F*(1, 15.454) = 1.533, *p* = 0.234, with no significant difference in contribution when they could punish (*M* = 7.224, *SE* = 0.356), compared to unable to punish (*M* = 6.598, *SE* = 0.359). The Gossip × Punishment interaction did not significantly predict contribution, *F*(1, 15.454) = 0.031, *p* = 0.862.

The effect of round number was marginally significant, *F*(3, 261) = 2.621, *p* = 0.051. Contribution increased from round 1 to round 2, then decreased (*M*s = 6.981, 7.090, 6.999, 6.574). Pairwise comparisons with Bonferroni correction revealed only a significant difference in cooperation between round 2 (*M* = 7.090, *SE* = 0.270) and round 4 (*M* = 6.574, *SE* = 0.287) (*SE* = 0.192, *p* = 0.047). There was also a significant Punishment × Round Number interaction, *F*(3, 261) = 4.212, *p* = 0.006. Further paired comparisons revealed no significant effect of punishment on cooperation in round 1 (*p* = 0.800) and round 2 (*p* = 0.349), but participants tended to be more cooperative when they could punish (vs. could not punish) in round 3 (*SE* = 0.540, *p* = 0.059, and in round 4 (*SE* = 0.573, *p* = 0.081). The Gossip × Round Number interaction (*p* = 0.562) or the Gossip × Punishment × Round Number interaction (*p* = 0.589) were not significant (Fig. S6).

### Gossip versus punishment: Efficiency in promoting individual earnings

Figure 1b shows participants’ total earnings across the four-round PGG in each condition. We conducted a linear mixed model (LMM) analysis with participants’ total earnings in the PGG as dependent variable, and gossip and punishment as between-subjects factors. The variance across sessions was modeled with a random intercept^32^. We found a significant effect of gossip, *F*(1, 15.993) = 13.879, *p* = 0.002, with more total earnings of points when participants could gossip (*M* = 64.274, *SE* = 1.587), compared to unable to gossip (*M* = 55.909, *SE* = 1.588). The effect of punishment was also significant, *F*(1, 15.993) = 19.135, *p* < 0.001, with less total earnings of points when participants could punish (*M* = 55.180, *SE* = 1.585), compared to unable to punish (*M* = 65.003, *SE* = 1.591).

These main effects were qualified by a significant Gossip × Punishment interaction, *F*(1, 15.993) = 4.878, *p* = 0.042. Further paired comparisons revealed that participants earned significantly fewer points in the punishment condition than in the control condition (mean difference = 14.782, *SE* = 3.177, *p* < 0.001). However, their earnings in the gossip-and-punishment condition was significantly higher than the punishment condition (mean difference = 13.325, *SE* = 3.169, *p* = 0.001), but did not differ from the gossip condition (mean difference = 4.863, *SE* = 3.174, *p* = 0.145). These results suggest that gossip can counteract the negative effect of punishment on efficiency and enhance individual welfare.

### Gossip versus punishment in maintaining cooperation

The mean levels of trust (number of points participants sent to the responder as an investor) and trustworthiness (percentage of points participants returned to the investor as the responder) in the TG in each condition are presented in Fig. 1c,d. To test the behavioral effects of removing gossip and punishment, we conducted linear mixed model (LMM) analyses on trust and trustworthiness, with gossip and punishment as between-subjects factors. The variance across sessions was modeled with a random intercept^32^. Gossip had a significant effect on trust, *F*(1, 16.587) = 6.050, *p* = 0.025, with more behavioral trust in response to the initial option to gossip (*M* = 7.910, *SE* = 0.432), compared to no gossip (*M* = 6.407, *SE* = 0.432). However, the effect of punishment, *F*(1, 16.587) = 0.277, *p* = 0.606, or the Gossip × Punishment interaction, *F*(1, 16.587) = 0.262, *p* = 0.615, did not significantly predict trust.

The main effect of gossip on trustworthiness was also significant, *F*(1, 15.889) = 6.654, *p* = 0.020, with greater percentage of points returned when participants were initially able to gossip (*M* = 0.400, *SE* = 0.022), compared to unable to gossip (*M* = 0.319, *SE* = 0.022). The effect of punishment, *F*(1, 15.889) = 0.817, *p* = 0.380, or the Gossip × Punishment interaction, *F*(1, 15.889) = 0.341, *p* = 0.567, did not significantly predict trustworthiness. The significant main effects of gossip on trust (*p*s = 0.015 and 0.025) and trustworthiness (*p*s = 0.014 and 0.020) still existed after controlling for participants’ total earnings in the PGG or the order of acting as investor and responder. These results suggest that gossip can be more effective than punishment in maintaining trust and cooperation when these mechanisms are absent in future interactions.

## Discussion

The present research contributes to our understanding of the relative ability of gossip and punishment to promote and maintain cooperation. Although gossip can increase cooperation and deter selfish behavior^13^,^14^,^30^, and is an effective and low-cost form of punishment^33^, the present research goes beyond these findings by directly comparing gossip and punishment in a behavioral experiment. We found that (a) people were more cooperative in response to potential gossip, and that (b) punishment tended to increase cooperation in the last two rounds of the PGG, but did not have a positive overall effect. Indeed, our findings on punishment are consistent with recent evidence that punishment did not increase defectors’ subsequent cooperation^34^. The mere option to gossip about each other was also relatively more effective than punishment itself in promoting cooperation across the four-round PGG, as revealed by the effect sizes of the gossip condition and the punishment condition, each separately compared to the control condition (*d* = 0.380 versus *d* = 0.169). Gossip also increased participants’ earnings and enhanced efficiency, whereas punishment significantly decreased participants’ earnings across the four-round PGG. More importantly, people earned more in the gossip-and-punishment situation than the situation with only punishment. This suggests that the opportunity to gossip and share information could counter the negative effect of punishment on efficiency and thus increase social welfare. Finally, we found that gossip makes people more likely to trust others and be trustworthy during future interactions without opportunities to gossip, whereas punishment did not influence trust and trustworthiness after its removal. Overall, our findings suggest that gossip can be relatively more efficient and effective than punishment to promote and maintain cooperation when gossip involves no cost whereas punishment is moderately costly.

Our results add to the literature on reputation and punishment in promoting cooperation^2^,^4^,^15^,^29^. Consistent with previous research on the interaction of indirect reciprocity and costly punishment^4^, we found that the extra option to gossip significantly increases the cooperative efficiency of punishment. More importantly, our research extends previous findings by testing whether gossip could maintain cooperation after its removal, compared to costly punishment. That punishment did not affect trust and trustworthiness in the TG seems at odds with previous evidence of a decrease in cooperation after punishment is removed^2^,^27^,^28^,^29^. There are at least two possible reasons for this inconsistent finding with previous research. First, we found that punishment had no significant overall effect on raising levels of cooperation and this “gain” in cooperation may have been too small to cause a substantial decline in cooperation. Our research differs from previous work that used a centralized punishment mechanism with explicit rules^27^, which might induce greater initial levels of cooperation than the peer punishment we used. Alternatively, people may learn to adjust their behavior gradually in response to peer punishment, and we only had participants interact for four rounds in the PGG. Second, our experiment only involved two interactions in the TG after punishment was removed, while prior research involved more interactions after its removal^2^,^28^. Despite this, compared to the control condition, participants in the punishment condition displayed a decline in cooperation from the 4^th^-round PGG to the TG (when punishment was removed) (Fig. S6). More importantly, we found that gossip and reputation could maintain trust and cooperation even after the removal of potential gossip. Thus, the initial gossip option countered the negative effects of punishment on trust and cooperation when these two options were combined. This benefit of gossip was present when both gossip and punishment were available as well as after their removal. Our results suggest that, compared to the extrinsic incentive of punishment, concerns about one’s reputation in response to gossip can encourage people to orient their behavior toward obtaining the long-term mutual benefits of cooperation, rather than the short-term temptation to defect.

It is clear that finding effective and efficient solutions to resolving social dilemmas is an enduring challenge to scientists and practitioners. Hundreds of studies have uncovered the effectiveness of punishment, and one important point is that the mere availability of punishment promotes cooperation quite effectively^1^,^2^,^35^. In contrast, while gossip is natural in our evolutionary history (e.g., in hunter-gatherer societies), modern times (e.g., through social media), and in various societies^36^, it has received very little attention. This is surprising because effective and (especially) efficient solutions to social dilemmas at least in part seem to be rooted in reputation^3^.

Indeed, the present findings add credence to the power of gossip, and by implication, reputation in social dilemmas. While gossip may increase the effectiveness of punishment in promoting cooperation and efficiency, the present findings uncovered that gossip by itself may be a powerful “tool” to promote trust and cooperation. By and large, punishment becomes superfluous when gossip is present and salient, at least when gossip involves no cost whereas punishment is moderately costly. What is the broader meaning of these novel findings? In particular, how can societies, small and large, use gossip in a way that helps us – the people, policy makers, and politicians alike – to effectively resolve social dilemmas? We see two broad implications.

First, the above analysis on the benefits of gossip suggests the importance of self-regulatory capacity of relatively small communities, where gossip and reputation are often conveyed face-to-face or through informal networks. It is exactly these small communities that are able to coordinate and communicate, and relate solutions to local circumstances^37^,^38^. It is possible (if not likely) that smaller groups overcome many social dilemmas through low-cost strategies such as gossip and reputation, rather than costly punishment^23^. Punishment requires costly and time-consuming monitoring of others’ behavior, whereas gossip can facilitate people to monitor others’ behavior with lower cost. Indeed, our research suggests that people are less prone to costly punish others when given both options to gossip and punish (see Supplementary Information), which is consistent with previous evidence on the decreased use of costly punishment in situations with opportunities for reputation building and indirect reciprocity^4^. Thus, when people are able to convey norms and monitor each other’s behavior through reputation spreading, costly punishment is less needed, unless gossip turns out to be ineffective.

Second, punishment is often material and extrinsic, and may change behavior only in the short term. In contrast, gossip is less material and less short-term, but that is perhaps why it is so powerful. Gossip involves reputational consequences that extend over time, are enduring, and in many ways escape from one’s own control. These are features that, if anything, should make people especially vigilant of gossip and its effect on their reputation. Gossip can spread through multiple channels that one cannot foresee, and it can reach those in power and with more social connections, and perhaps most importantly, can create a reality that an uncooperative reputation is difficult to correct and may limit future interaction opportunities. Therefore, people may be motivated to gain (and maintain) a good reputation, and gossip and reputation systems could have a “sustainable effect” on cooperation and potentially cultivate voluntary cooperators.

The present research is among the first to examine (and compare) both gossip and punishment. Hence, it is important to acknowledge limitations and outline avenues for future research. First, our experiment involved real-time interactions among online participants. This design enabled us to observe behavioral dynamics in response to the options to gossip and punish and after these options were removed. However, due to unexpected attrition and low levels of actual log-ins during the experiment, we had to use a standard experimenter strategy (i.e., the experimenter took the participant role, and acted the same across conditions) whenever necessary. Unfortunately, the use of this strategy made it impossible to examine group efficiency (i.e., group earnings in each round) and potentially introduced some noise in the data. Nevertheless, we found the same results after including the experimenter strategy data in the analysis (For additional analyses see Supplementary Information). This evidence suggests that the experimenter strategy did not influence the validity of our results. Importantly, the online environment guarantees anonymity during interactions that is usually not easy to administer in the labs with small groups. Our results suggested the short-term “sustainable effect” of gossip in maintaining cooperation, and there is need for future research to investigate the long-run benefits of gossip over a longer time horizon. This can be realized through (a) three different periods of PGG, (i.e., period 1 with no option to gossip or punish, period 2 with the option to gossip or punish, and period 3 where the option to gossip or punish is removed, and (b) more rounds of interactions in each period.

Second, in our research paradigm, we treated gossip with no financial cost and punishment to involve moderate cost (i.e., fee-to-fine ratio of 1:3). Generally, this is a suitable manipulation that permits us to test our predictions, because gossip is essentially (at least financially) free^30^,^33^, whereas punishment is often costly^2^,^6^,^7^. Results from a meta-analysis suggest that while the fee-to-fine ratio of 1:3 is most commonly used, cost-to-fine ratios do not moderate the effect of punishment on cooperation^1^. Future research may examine potential boundary conditions on when gossip and punishment can promote (and maintain) cooperation (e.g., more or less costly gossip and punishment, different group sizes, and face-to-face vs. online communication). Moreover, participants were not able to exclude free riders or choose their partners based on the received notes, and previous research shows that gossip is more effective in promoting cooperation if people can select partners and ostracize free riders^30^. In fact, a recent agent-based simulation also suggests that gossip-based partner selection increases cooperation, whereas the strategy to defect after knowing about free riders’ reputation decreases cooperation^18^. Future research would benefit by examining how cooperation rates change in response to different gossip-based strategies. In particular, after receiving negative gossip about free riders, people may avoid (or even ostracize) these free riders or defect against them.

Third, it is possible that the mere option to communicate accounted for the positive effects of gossip on cooperation. Indeed, previous research suggests that communication with one’s group members before or during social interactions can promote social norms and trust, and enhance cooperation^39^,^40^,^41^. Importantly, gossip is not direct communication with one’s group members, but is a form of indirect communication that enables people to share diagnostic information or evaluations about their group members with these members’ future partners. Thus, gossip helps people to keep track of others’ behavior prior to direct interaction with others. Consequently, the options to gossip about each other could (a) increase group members’ concerns about their reputation, (b) help third parties to learn about these members’ past behavior, and further adjust their strategies (e.g., to defect or to avoid interaction) when they interact with these people, and (c) convey group norms about what behavior is acceptable (e.g., participants sharing notes in this experiment stating “This person is great, donated the full amount”, “This person only donated half their amount. Probably kind of a jerk”, see Table S2 in Supplementary Information). Although gossip can be malicious or self-serving, leading to biased reputations, people may have developed adaptive strategies to assess gossip veracity from various situational cues (e.g., multiple sources of information)^42^,^43^,^44^. Thus, despite their different targets, both gossip and direct communication may promote trust and cooperation, but the underlying mechanisms may vary — an important topic for future research.

To conclude, gossip, and more broadly reputation systems, may be easily overlooked as an efficient solution to promote cooperation and overcome social dilemmas. Indeed, humans closely monitor and evaluate each other’s behavior, and willingly share their evaluations with other third parties. To organize groups and communities in such a way as to promote gossip and reputation spreading, while aversive in many ways, may paradoxically help groups to reach cooperation now and in the future. While punishment may be effective in promoting cooperation, gossip is both effective and efficient as it brings about very little cost or externalities. Because the present findings underline the benefits of gossip, it may be advisable to consider gossip as a motivational solution to promote and maintain cooperation. Importantly, although our findings do suggest that punishment cannot really do it alone, future research is needed to investigate the boundary conditions of when gossip and punishment can most effectively and efficiently promote and maintain cooperation.

## Methods

### Participants and design

A total of 265 American participants (116 women, *M*_age_ = 32.05 years, *SD* = 9.93) recruited from MTurk voluntarily participated in the experiment. This exceeds the required sample size of 171 calculated by G*Power to achieve a statistical power (1-β) of .90 to detect a medium effect size (*f* = 0.25)^45^. Participants were paid US$2.00 as baseline payment and earned an extra bonus (US$1.60 at maximum) based on their decisions during the experiment. They were assigned to one of the four conditions of a 2 (no gossip vs. gossip) × 2 (no punishment vs. punishment) between-subjects design. We conducted a total of 20 experimental sessions, with 16 participants planned in each session. Due to a lack of log-ins and unexpected dropouts (some potentially due to network connection problems), we employed a standard experimenter strategy for missing participants (For further details see Supplementary Information). The experimental procedures were approved by the Scientific and Ethical Review Board at Vrije Universiteit Amsterdam (VCWE-2014-078). The experiment was carried out in accordance with the approved guidelines. Informed consent was obtained from all participants.

### Procedure

**Figure 2:**
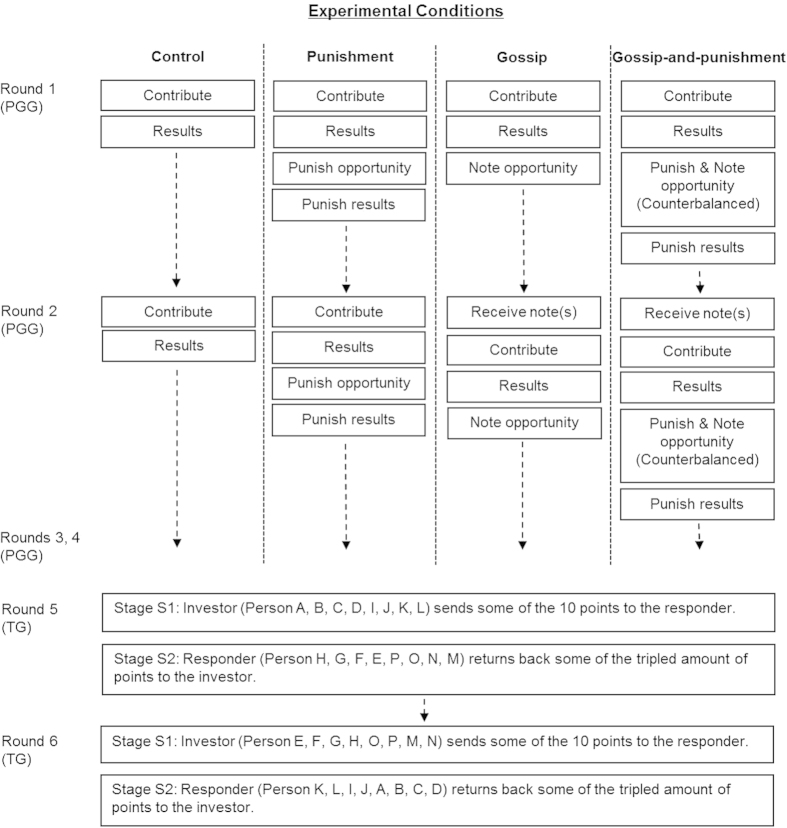
Procedure of the experiment. In each round of PGG, control condition involves only the contribution stage (S1), punishment condition involves the contribution stage (S1) and punishment stage (S3), gossip condition involves the contribution stage (S1) and gossip stage (S2), gossip-and-punishment condition involves the contribution stage (S1), gossip stage (S2), and punishment stage (S3).

We conducted the experiment through the Software Platform for Human Interaction Experiments (SoPHIE) connected with MTurk^46^. SoPHIE enables real-time social interactions between multiple participants online. Participants completed a four-round public goods game (PGG) and a subsequent two-round trust game (TG) (Fig. 2). They interacted with different partners in each round to guarantee no repeated interactions with the same partner, and this excluded potential retaliation or direct reciprocity (Table S1). Participants read about the procedure and answered several comprehension check questions before they started making decisions.

### Public goods game (PGG)

The PGG involved four participants in each group, with four groups in total. Each round consisted of a contribution stage (S1), a gossip stage (S2), and a punishment stage (S3). In stage S1, each participant was endowed with 10 points (US$0.1) and contributed simultaneously any amount (range: 0 to 10 points) to the group account and kept the remained points to their private account^2^. Total contributions to the group account were then doubled and divided equally among all group members (Fig. S3). Next, they were informed of each member’s contribution and earnings. The control condition with no option to gossip or punish only involved this contribution stage ended with feedback in each round.

In stage S2, we manipulated gossip by giving participants the option to send any (0–3) note(s) about other group member(s) (Fig. S4). These notes were then presented to those others’ new group members in the beginning of the next round^30^. In stage S3, we manipulated punishment by giving participants the option to assign deduction points (0–5) to each group member. Each deduction point they assigned to others cost them one point but decreased others’ earnings by three points (Fig. S5)^2^. Participants only learned about the total number of deduction points they received, without knowing who assigned the points. If their earnings in one round became negative after stage S3, this amount was deducted from their earnings in the other rounds. In the gossip-and-punishment condition, half of the participants had the option to gossip first, then punish in each round, and the other half had the option to punish first, and then gossip in each round.

### Trust game (TG)

The TG involved an investor and a responder. Across the two-round TG, half of the participants first acted as an investor, then as a responder, while the other half first acted as a responder, then as an investor^47^. They were paired with different partners in each round and made their decision in dyads. Each round involved two stages. In stage S1, the investor was endowed with 10 points (US$0.1) and could send any amount (range: 0 to 10 points) to the responder and keep the remainder for self. Only the amount sent to the responder was tripled. In stage S2, the responder could return some of the tripled amount back to the investor. The number of points investors sent to the responder was the measure of trust behavior. We used the return ratio (the amount the responder returned divided by the tripled amount received from the investor) as the measure of trustworthiness^48^.

For exploratory purposes, we measured participants’ social value orientation using the triple dominance measure of social values at the end of the experiment^49^. Because this is not related to our major objectives, we did not report the results related to this measure.

## Additional Information

**How to cite this article**: Wu, J. *et al*. Gossip Versus Punishment: The Efficiency of Reputation to Promote and Maintain Cooperation. *Sci. Rep*. **6**, 23919; doi: 10.1038/srep23919 (2016).

## Supplementary Material

Supplementary Information

Supplementary Table S2
