# Supplementary material for: Gossip Versus Punishment: The Efficiency of Reputation to Promote and Maintain Cooperation
Source: Sci Rep. 2016 Apr 4;6:23919. doi: 10.1038/srep23919 (PMC4819221; doi:10.1038/srep23919)
Supplement: Supplementary Information [file srep23919-s1.pdf]

Supplementary Information:

Gossip Versus Punishment:

The Efficiency of Reputation to Promote and Maintain Cooperation

Junhui Wu[a,*], Daniel Balliet[a], Paul A. M. Van Lange[a]

[a]Vrije Universiteit Amsterdam
Department of Experimental and Applied Psychology
Van der Boechorststraat 1
1081 BT Amsterdam, the Netherlands

[*] Corresponding author: j.wu@vu.nl; wjhbnu2009@gmail.com

Experimental Setup

Participants recruitment and treatments

We conducted the experiment from January 22, 2015 to February 4, 2015. Before the planned experimental sessions, we posted a sign-up survey periodically on Amazon Mechanical Turk (MTurk) to recruit participants for the upcoming "Real Time Interaction Experiment". Participants were paid a reward of US$0.1 for sign-up. In the sign-up survey, they were asked to choose all the possible time slots (i.e., 11:00-11:30 EST, 14:00-14:30 EST, and 16:30-17:00 EST) in the upcoming two days when they were available for participating. Participants provided their Worker ID that was needed for further email invitation. They were asked to check their email over the next two days.

The periodical sign-up survey enabled us to build a participant panel for each experimental session that needed 16 participants. We sent three email notifications (one day before, one hour before, and right after the session HIT was posted on MTurk) to around 40 to 60 participants using Python (see Emailing Workers Using Python) before each session. The email notification included the **experiment HIT name**, **HIT posted time**, and **an access password for this HIT**. Participants were asked to make their decisions in a timely manner and also be patient when they had to wait for others during the experiment.

Our original plan was to recruit 256 participants for 16 sessions (16 participants per session). Although we managed to email far more participants than needed before each session to guarantee that all spots were taken, some problems occurred during the experiment. Problems include a lack of log-ins, unexpected dropouts, and network connection problems reported by participants. When this happened, we employed a standard experimenter strategy for missing participants ($n \leq 5$ per session). That is, the experimenter took the participant role and (a) always

contributed 5 points in the public goods game, (b) did not gossip or punish when there was a chance, (c) always sent 5 points as an investor, and (d) returned some points to achieve equality in the trust game, or returned 0 points if equality was impossible. We terminated a (failed) session whenever there were too many dropouts ($n > 5$), and paid participants in these sessions the baseline payment of US$2 for participation.

Experimental platform and interface screens

We conducted the experiment through the Software Platform for Human Interaction Experiments (SoPHIE) connected with Mturk. SoPHIE is a web-based platform to run real-time behavioral experiments[1]. Below are the experimenter screens and participant screens for different experimental conditions (Fig. S1-S5):

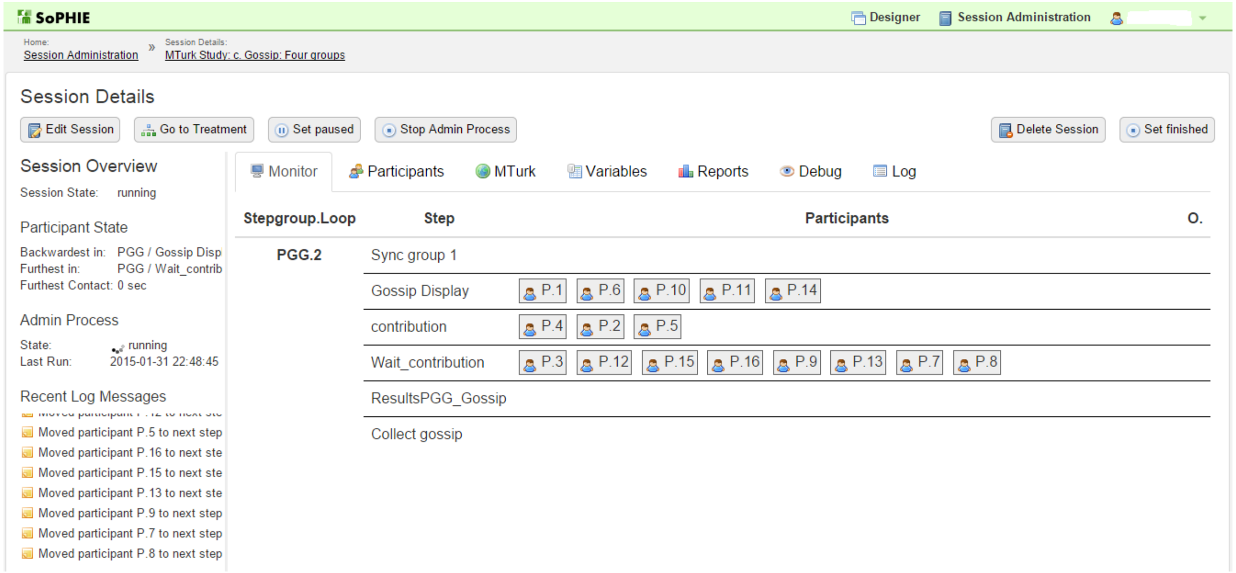

Figure S1. The session administration page of the experimenter screen (gossip condition with only gossip option)[1]. Permission to use the digital images of the SoPHIE logo and the screenshots has been granted by SoPHIE Labs, a division of Symbic GmbH, Osnabrück, Germany.

SoPHIE

Instructions

Welcome!
You are about to participate in a decision-making experiment. The experiment has 16 participants and consists of two parts (6 periods in total). Besides the $2 for participation, you have an opportunity to earn extra points based on you and others' decisions in each period. At the end of the experiment, you will receive feedback about the total amount of points you earn. Each point is worth money. The sum of points you earn in all periods will be converted to money based on the exchange rate

$1 = 100 points.

You will be paid the total amount (i.e., $2 plus extra money) through MTurk within two days after the experiment only if you have finished the experiment.

NOTE: Your identity will never be made known to any other participants at any time. Each participant will receive a capital letter to represent them during the experiment.

Please read the instructions carefully (you will have to answer some questions after the instructions), and do not switch to other unrelated tasks once you start. Otherwise, other participants will have to wait for you. Please respect others' time and also be patient when you have to wait for others when they are making their decisions!

Continue ...

Figure S2. The first-page instructions on participants' screen after they logged in (all conditions)[1]. Permission to use the digital images of the SoPHIE logo and the screenshots has been granted by SoPHIE Labs, a division of Symbic GmbH, Osnabrück, Germany.

SoPHIE

Part 1

Period 1: The members in your group in Period 1 are **Person A, B, C, D** .
Please keep in mind that you are **Person A** throughout the experiment.

Now each of you receives **10 points**. You have to decide **at the same time** how many of these 10 points to contribute to the group account and how many to keep for yourself. You and other group members can each choose any amount between 0 and 10 points to contribute.

The number of points contributed to the group account will be doubled and divided equally among all the four group members. That is, for each point contributed to the group account, each group member receives 0.5 point. Points not contributed will belong to yourself.

Your payoff in this period consists of (1) the points you keep for yourself and (2) your earning from the group account:

Payoff in this period = (10 - contribution) + (0.5 × sum of contributions)

After you have made your decisions, you will be informed of the decisions made by other group members and their earnings in this period.

Please enter the number (0-10) of points you contribute to the group account: Submit ...

Figure S3. Instructions on participants' screen in the contribution stage S1 (control condition with no gossip or punishment option)[1]. Permission to use the digital images of the SoPHIE logo and the screenshots has been granted by SoPHIE Labs, a division of Symbic GmbH, Osnabrück, Germany.

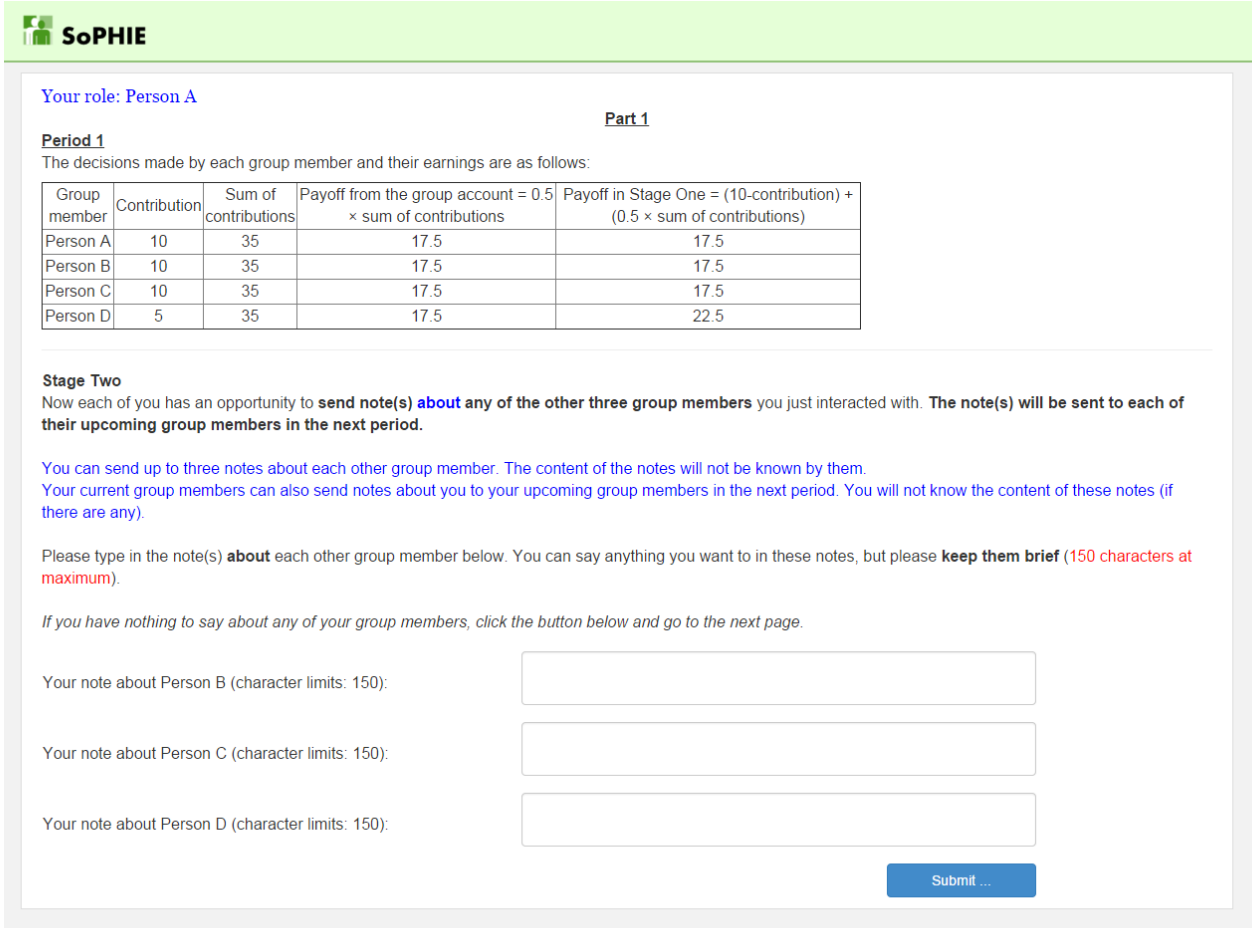

SoPHIE

Your role: Person A

Part 1

Period 1

The decisions made by each group member and their earnings are as follows:

Group member	Contribution	Sum of contributions	Payoff from the group account = 0.5 × sum of contributions	Payoff in Stage One = (10-contribution) + (0.5 × sum of contributions)
Person A	10	35	17.5	17.5
Person B	10	35	17.5	17.5
Person C	10	35	17.5	17.5
Person D	5	35	17.5	22.5

Stage Two

Now each of you has an opportunity to **send note(s) about any of the other three group members** you just interacted with. **The note(s) will be sent to each of their upcoming group members in the next period.**

You can send up to three notes about each other group member. The content of the notes will not be known by them.
Your current group members can also send notes about you to your upcoming group members in the next period. You will not know the content of these notes (if there are any).

Please type in the note(s) **about** each other group member below. You can say anything you want to in these notes, but please **keep them brief** (150 characters at maximum).

If you have nothing to say about any of your group members, click the button below and go to the next page.

Your note about Person B (character limits: 150):

Your note about Person C (character limits: 150):

Your note about Person D (character limits: 150):

Submit ...

Figure S4. Instructions on participants' screen in the gossip stage S2 (gossip condition with only gossip option)[1]. Permission to use the digital images of the SoPHIE logo and the screenshots has been granted by SoPHIE Labs, a division of Symbic GmbH, Osnabrück, Germany.

SoPHIE

Your role: Person A

Part 1

Period 1

The decisions made by each group member and their earnings are as follows:

Group member	Contribution	Sum of contributions	Payoff from the group account = 0.5 × sum of contributions	Payoff in Stage One = (10 - contribution) + (0.5 × sum of contributions)
Person A	0	20	10	20
Person B	0	20	10	20
Person C	10	20	10	10
Person D	10	20	10	10

Stage Two

Now each of you must decide how many deduction points to assign to each of the other three group members. You can assign **between 0 and 5 deduction points** to each other group member.

For each deduction point that you assign to a group member, it costs you 1 point, and decreases his/her payoff by 3 points.

Example:

- *If you assign **0 deduction points** to a group member, his/her payoff will **not** be changed by you.*
- *If you assign **2 deduction points** to a group member, it will cost you **2 points**, and decrease his/her payoff by **6 points**.*

Meanwhile, **your payoff will be reduced by 3 points for each deduction point that is assigned to you by other group members.**

Example:

- *If **no one** assigns deduction points to you, your payoff will **not** be changed by others.*
- *If a group member assigns **2 deduction points** to you, it costs this person **2 points**, and decrease your payoff by **6 points**.*

Thus, you will lose points in Stage Two if you assign deduction points to others or others assign deduction points to you.

Your total payoff in this period is calculated as follows:

Total payoff = Payoff in Stage One - sum of deduction points assigned by you - (3 × sum of deduction points assigned to you)

Note: Your total payoff in this period can be negative, if the points you lose in Stage Two exceed your payoff in Stage One. When this happens, the losses will be taken from the points you earn in other periods of the experiment.

* required field.

You are Person A. Please fill in the number of deduction points you assign to your group members. This number should be between 0 and 5 (including 0 and 5).

Number (0 to 5) of deduction points you assign to Person B:*

Number (0 to 5) of deduction points you assign to Person C:*

Number (0 to 5) of deduction points you assign to Person D:*

Submit ...

Figure S5. Instructions on participants' screen in the punishment stage S3 (punishment condition with only punishment option)[1]. Permission to use the digital images of the SoPHIE logo and the screenshots has been granted by SoPHIE Labs, a division of Symbic GmbH, Osnabrück, Germany.

Grouping

Participants interacted with different partners in each round of the public goods game (PGG) and the trust game (TG) to guarantee no repeated interactions with the same partner (Table S1).

Table S1. Participant grouping across the four-round PGG and two-round TG

Round	Grouping
PGG1	Group 1-4: ABCD, EFGH, IJKL, MNOP
PGG2	Group 1-4: AEIM, BFJN, CGKO, DHLP
PGG3	Group 1-4: AFKP, BELO, CHIN, DGJM
PGG4	Group 1-4: AGLN, BHKM, CEJP, DFIO
TG1	Investor/Responder: A/H, B/G, C/F, D/E, I/P, J/O, K/N, L/M
TG2	Investor/Responder: E/K, F/L, G/I, H/J, O/A, P/B, M/C, N/D

Note: The sixteen participants numbered as P.1 to P.16 were given the label of Person A to Person P during the experiment.

Additional Analyses

Correlation between being punished and subsequent change in contribution

We computed the difference in contribution between (a) round 2 and round 1 (Δ1), (b) round 3 and round 2 (Δ2), and (c) round 4 and round 3 (Δ3). Then we calculated their correlations with the total deduction points one received in round 1, 2, and 3 when participants had the option to punish. We found that being punished in a previous round makes participants more likely to increase (and less likely to decrease) contribution in the next round, rs(133) = 0.483, 0.364, and 0.344, ps < 0.001.

Experimental results including the data of experimenter strategy

To test whether the use of experimenter strategy influenced our results. We used linear mixed model (LMM) analyses to fit the data (including the data of experimenter strategy) with (a)

average contribution in the PGG, (b) total earnings in the PGG, (c) trust, and (d) trustworthiness in the TG as dependent variables. We included gossip and punishment as between-subjects factors and modeled the variance across sessions with a random intercept[2].

For the average contribution in the PGG, the main effect of gossip was marginally significant, $F(1, 16) = 4.011$, $p = 0.062$, with more average contribution when participants could gossip ($M = 7.023$, $SE = 0.307$), compared to unable to gossip ($M = 6.153$, $SE = 0.307$). However, the main effect of punishment, $F(1, 16) = 1.399$, $p = 0.254$, or Gossip × Punishment interaction, $F(1, 16) = 0.001$, $p = 0.975$, did not significantly predict average contribution.

For the total earnings in the PGG, gossip had a significant main effect, $F(1, 16) = 13.311$, $p = 0.002$, with more total earnings of points when participants could gossip ($M = 64.844$, $SE = 1.663$), compared to unable to gossip ($M = 56.263$, $SE = 1.663$). There was also a significant effect of punishment, $F(1, 16) = 16.464$, $p = 0.001$, with less total earnings of points when participants could punish ($M = 55.781$, $SE = 1.663$), compared to unable to punish ($M = 65.325$, $SE = 1.663$), The Gossip × Punishment interaction was also significant, $F(1, 16) = 4.806$, $p = 0.043$. Further paired comparisons revealed that participants earned significantly fewer points in the punishment condition than in the control condition (mean difference = 14.700, $SE = 3.326$, $p < 0.001$). Their earnings in the gossip-and-punishment condition was significantly higher than the punishment condition (mean difference = 13.738, $SE = 3.326$, $p = 0.001$), but did not differ from the gossip condition (mean difference = 4.387, $SE = 3.326$, $p = 0.206$).

For the level of trust, there was a significant effect of gossip, $F(1, 16) = 5.350$, $p = 0.034$, with more behavioral trust in response to the initial option to gossip ($M = 7.394$, $SE = 0.375$), compared to unable to gossip ($M = 6.166$, $SE = 0.375$). The main effect of punishment, $F(1, 16)$

= 0.195, *p* = 0.665, or the Gossip × Punishment interaction, *F*(1, 16) = 0.137, *p* = 0.716, did not significantly predict trust behavior.

For the level of trustworthiness, there was also a significant effect of gossip, *F*(1, 15.704) = 6.923, *p* = .018, with more percentage of points returned when participants could initially gossip (*M* = 0.401, *SE* = 0.019), compared to unable to gossip (*M* = 0.328, *SE* = 0.020). The main effect of punishment, *F*(1, 15.704) = 0.876, *p* = 0.364, or the Gossip × Punishment interaction, *F*(1, 15.704) = 0.630, *p* = 0.439, were not significant.

Tendency to punish others and be punished by others

Here we examined participants' tendency to punish others (i.e., deduction points they assigned to others) and be punished by others (i.e., deduction points assigned to them) when there was an option to punish. We used linear mixed model (LMM) analysis to fit the data from the conditions with punishment option, with gossip as a between-subjects factor, round number as a repeated measure with an unstructured covariance matrix. The variance across sessions was modeled with a random intercept[2]. Participants were less likely to punish others in each round when they could also gossip (*M* = 0.491, *SE* = 0.197), compared to unable to gossip (*M* = 1.213, *SE* = 0.195), *F*(1, 8.202) = 6.787, *p* = 0.031. This tendency to punish others in response to gossip option was consistent across rounds, as revealed in a nonsignificant Gossip × Round Number interaction, *F*(3, 133) = 0.085, *p* = 0.968. Participants were also less likely to be punished in each round when there was an extra option to gossip (*M* = 0.350, *SE* = 0.137), compared to no gossip option (*M* = 0.972, *SE* = 0.135), *F*(1, 8.300) = 10.467, *p* = .011, and this tendency to be punished in response to gossip option was consistent across rounds, as revealed in a nonsignificant Gossip × Round Number interaction, *F*(3, 133) = 0.208, *p* = 0.890.

Tendency to gossip about others and be gossiped by others

Here we examined participants' tendency to gossip about others (i.e., number of notes they sent about others) and be gossiped by others (i.e., number of notes others sent about them) when there was an option to punish. We used linear mixed model (LMM) analysis to fit the data from the conditions with gossip option, with punishment as a between-subjects factor, round number as a repeated measure with an unstructured covariance matrix. The variance across sessions was modeled with a random intercept[2]. Participants did not differ in their tendency to gossip about others when they could not punish (M = 2.306, SE = 0.151) and when they could also punish (M = 2.179, SE = 0.151), $F(1, 7.979) = 0.353$, $p = 0.569$. Round number significantly predicted gossip tendency, $F(3, 131) = 8.331$, $p < 0.001$. Pairwise comparisons with Bonferroni corrections revealed that gossip tendency was higher in round 2, 3, and 4, compared to round 1 ($p = 0.001$, $p < 0.001$, and $p = 0.002$), but did not differ across round 2, 3, and 4 (ps > 0.10). Participants also did not differ in their tendency to be gossiped by others when they could not punish (M = 1.960, SE = 0.102) and when they could also punish (M = 1.715, SE = 0.102), $F(1, 8.164) = 2.897$, $p = 0.126$. Round number significantly predicted their tendency to be gossiped, $F(3, 131) = 3.247$, $p = 0.024$, with a higher tendency to be gossiped in round 2 (SE = 0.105, p = 0.066) and round 3 (SE = 0.097, p = 0.023), compared to round 1.

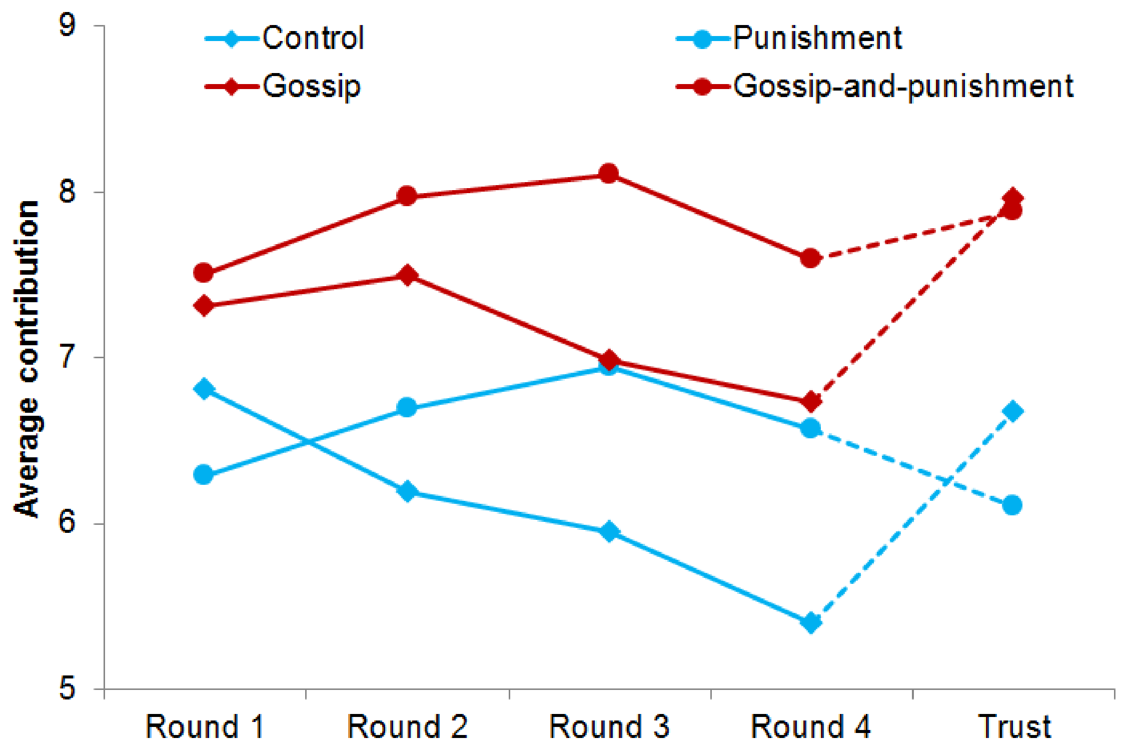

Figure S6. Average contribution in each round of the PGG and trust in the TG across the four conditions of a 2 (Gossip) × 2 (Punishment) between-subjects design.

Notes Sent by Participants in the Conditions with Gossip Options

We categorized almost all the notes sent by participants as gossip that involved (a) diagnostic information about group members' behavior and/or (b) positive or negative evaluations about these others. We did not categorize as gossip messages like "love" (3), "hi" (3), "mmmmM" (3), or "Why do we even have to leave notes" (1) that was sent by three participants in the first two rounds. All the gossip notes sent by participants across the four-round PGG in the conditions with gossip option are presented in the table below (Table S2). Each row represents the notes about a person's behavior (including the experimenter strategies) sent by different group members across the four-round PGG.

Table S2. The notes sent by participants who had the option to gossip (gossip condition: session S3, S8, S13, S16, and S19; gossip-and-punishment condition: session S5, S6, S7, S15, and S18)

Session	1st Round PGG	2nd Round PGG	3rd Round PGG	4th Round PGG
	[Good contributor]; [Person A contributed all 10 points; 3/4 of total group contributed 10, 1/4 contributed 5]; [contribute all 10 points]	[Contribution = 10]; [R1: 10 R2: 10]	[A sucks and is greedy.]	[Sent 5.]; [person donated 11 points, dont know how they got 11 points]
	[Person J contributed 4.]	[go bengals!]; [Sent 6]; [donated 6 most from group]	[Contribution = 0]; [contributed 0]	[Sent 0 all 3 times]; [0 ALL ROUNDS]
	[Person K contributed 5.]	[Contributed 5 both rounds]	[Has done 5 in each round, what a dinkbag.]	[Contribution = 5]; [Sent 5]; [Gave half.]
		[contributed 6]; [Donates about half.]	[Sent all 10]; [R2: 5 R3: 10]	[Not a good dude. 2, 2, 0. Don't trust them.]; [person donated 11 points, dont know how they got 11 points]
	[sent all 10 points]	[Great person. Donated all both times.]; [R1: 10 R2: 10]	[gave 10]; [contributed 10]	[Sent all]; [Super-nice person, gave 10.]
	[Contribution = 6]	[gave 5]; Person B: Sent 5]; [donated 5 average for group]	[Only contributed 2 (probably after seeing me contribute 10 both times), everyone else	[Great person. Gave 9 in last round]; [Sent 9!]

S3			contributed 5. contributed 5 in both previous rounds]; [Not trust-worthy! Only gave 2/10!!!]	
	[Contribution = 5]; [sen5 5]	[Contributed 5 both rounds]	[Sent 5.]; [Sent half]; [R2: 5 R3: 5]	[contributed 5]
	[Contribution = 5]; [sent 5]	[Sent 5.]; [contributed 5]; [Donates about half.]	[Has done 5 in each round, what a dinkbag.]	[gave 5]; [Sent 5 all 3 times]; [5]
	[This person is great. Donated the full amount.]; [Person B contributed all 10 points; 3/4 of total group contributed 10, 1/4 contributed 5]; [contribute all 10 points]	[gave 5]; [go bengals!]; [donated 5 average for group]	[Sent 5.]; [R2: 5 R3: 5]	[Contribution = 5]; [Gave half.]
	[This person is great. Donated the full amount.]; [Good contributor]; [contribute all 10 points]		[person donated 11 points, dont know how they got 11 points]; [Gave half.]	[gave 0]
	[This person only donated half their amount. Probably kind of a jerk.]; [About half good in contributing]; [Person D contributed 5 points; 3/4 of total group contributed 10, 1/4 contributed 5]	[Sent 5.]; [Donates about half.]	[gave 5]; [Contribution = 5]	
	[Please treat this person nicely. They are very generous.]	[Great person. Donated all both times.]; [Contribution = 10]	[Only sent 4.]; [Sent 4]	[gave 10]; [Sent 10]
	[Round 1: contributed 5 points]; [This one's okay. Contributes half, but not all.]	[gave 5]; [go bengals!]; [Sent 5]	[Did 5, 5, 10, so I guess they're learning.]	[contributed 5]
	[Round 1: contributed 5 points]; [This one's okay. Contributes half, but not all.]	[Contributed 5 both rounds]	[gave 5]; [Contribution = 5]; [contributed 5]	[I think only 5's each round.]; [Sent 5.]; [person donated 11 points, dont know how they got 11 points]
	[Round 1: contributed	[Sent 5.]; [contributed	[person donated 11	[Contribution = 6];

	6 points]	5]	points, dont know how they got 11 points] [Contributed 5, same for all 3 rounds]	[Sent 6]
	[Person I contributed 5.]	[This is a superduche. They only donated 5 in each round.]; [Contribution = 5]; [R1: 5 R2: 5]	[person donated 11 points, dont know how they got 11 points] [Contributed 5, same for all 3 rounds]; [Gave half.]	[contributed 5]
**S8**		[Sent 9/10.]; [Was very fair]	[gave 9]; [gave 9]	[gave 9]; [donated 9]
	[Donated 10]; [good contributor]	[donated 10]; [gave all]	[donated 10]; [contributes all 10]	[gave 10]; [Contributed 10]
	[Very generous , team member donates all]; [good contributor]	[contributed the max]; [very generous]	[Contributed all 10]; [gave 10]	[gave 10]; [Contributed 10!]
	[Very generous , team member donates all]; [Donated 10]	[generous]; [Contributed 10]	[gave all]; [gave 10]; [Gave all!]	[Contributed all 10]; [donated all 10]
	[contributed 5 in the first round]; [fair]	[Only contributed 5 points]; [Contributed 5.]	[Gave 5]; [donated 5]; [only contributes 5]	[Sent 0. Be an ass to him.]; [gave only 5]; [Contributed 5!]
	[contributed 4 in the first round]; [unfair]	[Only donated 4]; [donated only 4]; [cheap]	[Donated 0, probably are here so they don't get fined]	[didn't contribute. Disappointing]; [gave 0 how annoying]; [donated 0]
	[generouse]	[Gave 10]; [very generous]	[donated all]; [gave 10]; [Gave all!]	[sent 10]; [gave nothing]
	[contributed 5 in the first round]	[submitted 10]; [Contributed 10]	[Contributed all 10]; [gave 10]	[Generous sent 10]; [Contributed 10]
	[They submitted all 10 points.]	[Very generous , team member donates all]; [gave all]	[donated all]; [gave all]; [Gave all!]	[Sent 10]; [Contributed 10!]
	[They only submitted 5 points.]	[Gave 5]; [still only contributed 5 even after seeing others contribute all]; [only contributes 5]	[Contributed 5!]	[Miser sent only 5]; [gave 5]; [Contributed 5]
	[They only submitted 3 points, kind of disappointing.]	[only submitted 5]; [generous]; [Contributed 5]	[Gave 8]; [only contributes 8]	[gave 8]
	[gave all points]; [Contributed 10 points]; [Contributed 10 points]	[Only contributed 5 points]	[donated all]; [gave all]; [gave 10]	[Generous sent 10]; [gave 10]
	[Sent 8/10 pts];	[Only donated 8];	[Contributed 9.];	[gave 8]; [sent 8]

	[Contributed 8 points]; [Contributed more than 6 points]	[donated 8]	[gave 9]; [gave 9]	
	[Sent all 10 points]; [gave all points]; [Contributed 10 points]	[Gave 10]; [contributed the max]	[Gave 10]; [donated 10]	[Contributed all 10]; [gave 10]
	[Sent all 10 points]; [gave all points]; [Contributed 10 points]	[submitted 10]; [generous]	[Generous]	[Sent 10]; [gave 10]
	[Only donated 5 out of his 10 , is playing smart]; [Donated 5]; [didn't contribute as much as the rest of us]	[0 points contributed!!! :( ]; [Didn't contribute anything.]	[Contributed 1! They are probably here so they don't get fined]	[gave zero! (was told gave 1 in the prior round)]; [sent 0]
	[only gave 5]	[only contributed 5]; [Paid half, apparently also did so in first round]	[plays it safe]; [Only gave 5 this time.]; [Only gave half]	[5]; [starting to get selfish]
	[Is generous.]; [generous]; [very generous]	[very generous]; [Donated 10]; [Generous]	[gave 10]; [10/10]	[Gave 10/10]; [Contributed all]
	[stingy]; [stingy]; [stingy]	[was stingy; the first round. contributed 8 2nd round]; [okay]	[Very generous team player]; [Gave all 10 this time.]; [Generous]	[Gave 10]
	[generous]; [Is generous.]; [very generous]	[Contributed 8 of their 10]; [gave 8]	[contributed 10]; [Gave 10]; [Contributed all]	[nice]; [contributed much]
	[didn't contribute at all]; [10 point donation]; [Contributed all]	[Is generous]; [Contributed everything in second round]	[gave all]; [gave 10]; [10/10]	[Very generous]; [Gave 10]
**S13**	[N only contributed 2; everyone else contributed 10]; [He is not going to donate a lot. 2 point donation]; [Only contributed 2]	[only paid 1]; [Not very generous at all, donated only 1]; [Only gave 1 to the group]		[naughty]; [3]
	[didn't contribute much]; [Contributed all]	[is generous]; [okay]	[8]; [Gave 8]; [Contributed 8]	[10]; [Generous]
	[contributed everything]; [10 point donation]	[10pt]; [gavw 10]	[Very generous team player]; [very generous];	[gave 10]; [Contributed all]

			[Generous]	
	[Is practical]; [only gave 6]	[paid 8]; [gave 8]; [Contributed 8]	[10]; [contributed 10]; [Contributed all]	[very generous]
	[Is generous]; [gave 10]	[is generous]; [Generous the first round. contributed 10 2nd round]; [generous]	[gave 5]	[gave 10]; [Gave 10/10]; [Contributed all]
	[Is generous]	[10pt]; [Contributed all 10]	[gave all]; [10/10]	[10 points]; [Generous]
	[Contributed to group]; [generous]	[Is generous]	[10]; [contributed 10]; [Gave 10]	[gave 10]; [Gave 10/10]
	[In first round, F contributed 10]; [generous]	[paid 10]; [very generous]; [Donated 10]	[Very generous team player]; [very generous]; [Gave all 10.]	[Stingy. only 1 point]; [1]
	[in first round, G contributed 10]; [Contributed to group]	[is generous]; [contributed 10 1st and 2nd round]	[gave all]; [gave 10]	[naughty]; [5]; [half]
	[In first round, H contributed 1 while everyone else contributed 10]; [Contributed little to the group]; [greedy]	[greedy]; [Doesn't seem to like to share, gave 5 this time.]; [gave 5 fucker]	[gave 5]	[plays it safe]; [only contributed 5]; [Gave only 5]
	[stingy]; [Plays it safe and halved the points.]; [stingy]	[Is practical]; [only contributed 5]; [Paid half, apparently also did so in first round]	[gave 5]	[Stingy. only 5 points]; [5]; [Gave half]
	[fair decision maker]; [contributed 5 points]	[fair, 5 points in second round]; [contributed half]; [Contributed five.]	[Donated 5.]; [gave 5]	[0 out of 10 must be a republican]
	[Made the least amount of money. What a Dummy.]	[nice elbows]; [put in 5 of 10]; [gave 5]	[fair, 4 points p3]; [below average generosity]; [4 out of 10 I have lot faith in humanity]	[contributed 5 points]; [gave 5]
	[Made the most money. Not a Dummy.]	[contributed 5 points]; [k contributed 5 points]	[The person was very fair]; [gave 5]	[fair]
			[cautious, but fair]; [fair]; [gave 5]	[Very Fair.]; [5 out of 10 the meek shall inherit the earth that is barren from the greed of the many]
	[contributed the least	[The person was very	[contributed half]; [5	[Very Greedy :(.];

S16	amount]	fair.]; [contributed half]; [Contributed five.]	out of 10 must be a libertarian]	[greedy, contributed nothing!]; [Did not contribute at all]
	[was generous in the first round, offered 7 out of 10 points]	[put in 3 of 10]; [gave 3]	[contributed 4 points]; [Donated 4]	[Generous]; [7 out of 10 a small fleeting ray of hope]
		[contributed 6 points]; [O contributed 6 points]	[fair]; [gave 6]	[gave 7]
			[The person was very fair]; [Donated 5.]; [gave 5]	[half]; [contributed 5 points]; [gave 5]
	[This person was very fair during the previous round.] [contributed 5 points]	[great eyes]; [gave 4]	[cautious, but fair]; [gave 5]	[Paid Half.]
	[This person was very fair during the previous round.]; [fair decision maker]	[beyond generous]; [C contributed 10 points]	[Did not contribute at all!]; [Donated Nothing, Butt made the most money. Stingy.]	[half]; [gave 5]
	[This person was very fair during the previous round.]; [fair decision maker]; [contributed 5 points]		[contributed half]; [fair, 4 pts p3]; [5 out of 10 the ultimate goal of humanity is to destroy itself]	[generous, gave more than half]; [gave 0]
	[gave 5]; [E contributed 5 points]	[The person was very fair]; [fair, 5 points 2nd round]; [Contributed five.]	[cautious, but fair]; [fair]	[less that half but made a little more]; [contributed 3 points]
	[very generous]; [F contributed 10 points]	[very generous]; [powerful speaking voice]; [contributed all 10]	[The person was very fair]; [Donated 5.]	
	[very generous]; [gave 10]	[beyond generous]; [contributed 10 points]	[very generous]; [generous, 10 points in period 3]; [above average generosity]	[Very Generous]
	[mediocre generosity]; [gave 5]; [H contributed 5 points]		[contributed 7 points]; [Donated 7]	[Paid Half.]; [fair]
		[The person was very fair]; [very generous, offered 8 points in second round]; [generous contributed 8]	[contributed 5 points]	[very generous, gave all 10]; [gave 0]
	[Good tipper]	[contributed the	[shared majority];	[added 9]; [Added 9]

S19		maximum amount. was the only player out of the 4 to do that.]; [good tipper]	[Good. Contributed 8/10]; [8 points]	
	[Kept most of the points]; [minimum contributed]	[6 points]	[contributed a healthy 8.]; [Added 8]	[Super generous. All in.]; [10 given]; [good]
	[contributed a lot of their points]; [good contribution]	[8 given]; [Added 8]	[gave 10]; [Super generous!]; [All points]	[contributed a healthy 8.]; [contributed most]
	[contributed a lot of their points]; [Shared most]	[Good, 8/10]	[good]	[gave 8]; [Added 8]
		[gave 6]; [good tipper]	[Contributed 7]; [Added 7]	[contributed majority]; [contributed most]
	[this person looked out for the team rather than himself/herself. this person appears to be unselfish and a good team player.]	[Contributed all of their points]; [All points]	[10 given]; [contributed all]	[gave 10]; [all in]; [Added 10]
		[shared half]; [bad tipper]; [Added 5]	[added 5]; [good]	[5 points]
		[added 8]	[gave 3]; [barely shared]; [Only 3 points, even though last time they gave more]	[Contributed all 10]; [10 given]; [good]
	[moderate giver]; [5]	[Contributed a moderate amount (5)]; [Half points]	[added 5]; [good]	[shared half]; [only contributed 5.]; [contributed 5]
	[moderate giver]	[gave all]; [Added 10]	[contributed most]	[Contributed 8]; [8/10]; [good]
	[Is generous]; [Good tipper]	[added 10]; [Super generous!]	[Contributed all 10]; [was the only person who contributed all 10.]; [Added 10]	[All points]
	[Added 3]	[gave 7]; [contributed 7.]	[all in]	[Contributed 9]; [Generous, 9/10]; [9 given]
	[seems like a nice person]; [Added 5]	[Contributed 6]	[gave 8]; [shared majority]; [Good. Contributed 8/10.]	
	[good]	[barely shared any]; [bad tipper]	[Contributed 5]; [only gave 5.]	[gave 0]; [put in zip]
	[good]; [Added 7]	[added 5]; [Not that generous 5/10]	[7 given]	[shared half]; [gave only 5.]

	[contributed a moderate amount of their points]; [Shared half]; [minimum contribution]	[gave 5]; [contributed only 5.]; [good tipper]	[5 given]; [contributed half]	[5 points]
	[contrib 6]	[contributed 7]; [contributed 7 out of 10]; [Generous contribution.]	[contributed 7]; [contributed 7]	[contrib 6]; [Contributed 7]
	[Didn't contribute anything in period 1]; [Did not contribute anything.]; [This person did not contribute to the group account. Be wary.]	[half points given]; [only contributes half]	[Contributed 5 points]; [Only gave half]; [Only contributes 5 points]	[gave 0]; [contributed 5]
	[Contributed 6.]; [This person was fair in their contribution.]	[contrib 0]; [Contributed 8 of 10 points]	[Contributed all 10]; [contributed 10]	[Contributed 8]; [8 of 10 points]; [Contributed 8]
	[contributed 8 in period 1]; [This person was generous in their contribution.]	[Contributed 6 points]; [Contributed 6]	[contributed 6]; [contributed 6]	[Contributed 6]; [Contributed 6]
		[Contributed 8]; [contributed 8 out of 10]; [Generous contribution.]	[Contributed all 10 points]; [Contributed all 10 points]	[contributed 10]; [contributed all points!]; [Contributed the maximum]
	[Sent half of points]	[half points given]; [only contributes half]	[gave 0]; [Contributed 5]; [Contributed half. They were fair.]	[Contributed 5]; [contrib 5]; [Contributed 5]
**S5**	[Sent half of points]	[contributed 5 points]; [contrib 0]; [Contributed 5 of 10 points]	[contrib 5]; [contributed half]; [contributed 5]	[Gave half]; [contributed 5]; [Contributed half.]
	[Sent half of points]	[sent 5]; [Contributed half]; [Contributed 5]	[Only contributed half]; [contributed 5]; [contributed 5]	[gave 0]; [contributed 5]
	[Only contributed half of their points towards the group]; [contrib 5]	[only contributed 6]	[contrib 6]; [contributed 6]	[contributed 10]; [Contributed all 10]; [Contributed the maximum]
	[Contributed all their points towards the group!]	[contributed 10 points]; [Contributed all of their points twice]	[Contributed the maximum]; [Very generous contribution.]	[contributed 0, thaaaanks so much, jerk]

	[contrib 6]	[sent 7]; [Contributed 7]	[Contributed 8 points]; [Contributed 8 points]	[contributed 5]; [Contributed half.]
	[Person E contributed all 10 points]; [Contributed the maximum]	[Contributed all their points]; [contributed all 10]; [Very generous contribution.]	[contrib 10! :)]; [contributed all 10]	[gave 10]
	[contributed maximum amount; team player]; [Person F contributed all 10 points]; [Contributed the maximum]	[team player, did all 10]	[Contributed 8]; [contributed 8]	[Gave 7]; [Generous contribution.]
	[contributed maximum amount; team player]; [Contributed the maximum]	[contributed 10 points]; [contrib 10]	[Contributed 10 points]; [Contributed all 10 points]	[Contributed all 10]; [cantrib 10! :)]
	[contributed maximum amount; team player]; [Person H contributed all 10 points]	[sent 10!]; [Contributed all 10 points both rounds]	[gave 10]; [Very generous contribution.]	[contributed 10]; [Contributed all 10]; [contributed all points!]
	[contributed 5 in period 1]; [Contributed 5]	[Contributed 6]; [contributed 6]; [contributed 6 out of 10]	[gave 0]; [Contributed 8]	[Gave all 10]; [contributed 10]
	[Very generous]	[Gave the full 10!]; [Donated everything. Good person.]	[Gave 10 for the win.]	[Contributed 10 points]; [Gave all 10!]; [10!]
	[Hm. Gave 5 on the first round, keep an eye on him.]; [Contributed 5 points]; [Only sent 5]	[Reportedly contributed 5 first round, second round did 10! Has seemingly reformed :D]; [Very generous, submitted 10]	[Honest individual, gives freely.]; [contributed all]; [full 10!]	[Generous, donated everything.]
	[Donated 10 points]; [Contributed 10 points]		[donated 10!]	[Gave all, generous]; [Donated 10]
	[Donated 10 points]; [Gave the full 10. Clearly a mensch.]	[contributed 7]; [Donated 7]	[Very generous, gave 10]; [Donated everything. Good person.]	[donated 10]; [Gave all 10!]; [10!]
	[Contributed full amount--is worth it to do the same to get the maximum gain.]	[donated full 10]; [Donated everything. Good person.]	[Donates 10 consistently. (according to the note). How nice!];	[Contributed ZERO. May the fleas of a thousand camels infest his armpits.]; [Didn't

S6			[contributed all]; [full 10!]	donate any! Selfish]; [Did not donate]
		[contributed 10]; [Very generous, submitted 10]	[Donated 10]	[donated 10]; [Contributed 10 points]; [10!]
	[Contributed half this time.]	[Only gave 5. The weenie.]; [donated 5]	[Contributed 5 points]; [Only gave 5...]; [Donated half. Fair.]	[half contributed]; [Only 5 points]
	[Contributed half this time.]	[Contributed 5 points]; [contributed half]; [Donated 5]	[only donated 5 :(]; [Greedy bastard only contributed 5.]; [Sent only 5....]	[Consistently donates 5.]; [contributes only 5]; [Only donated half.]
	[CONTRIBUTED 5]; [selfish]	[contributed 8]; [Reportedly contributed 5 first round, second round did 8. Is getting the hang of it.]	[Contributed 8 points]; [Donated 8. Good player.]	[Gave 8. Good, not great.]; [Donated 8]
	[Very generous]	[Gave all ten. Quality person.]	[They (and everyone else) gave 10. It was a good round!]; [Donated 10]	[Consistently donates 10.]; [Generous, donated everything.]
	[Very generous]	[Contributed 0 points, the jerk]; [Did not donate]	[Donated 0 both this time and the last (according to notes). Don't expect anything from them.]; [Don't believe his lies. So greedy.]; [Donated 0 this round and reportedly the other two, as well.]	
	[Great contributor]	[donated full 10]; [Gave the full 10!]	[Contributed 10 points]; [Very generous, gave 10]	[Consistently donates 10.]
	[Put all points into pool. Good person.]; [Great contributor]	[contributed 10]; [Has contributed 10 points reportedly for the first round, and now the second :3]; [Very generous, submitted 10]	[donated 10!]; [Contributed all 10.]	[all contributed]
	[Donated half of points. Fair.]; [Great contributor]	[Mr. Indecisive here gave 7... okay I suppose.]; [donated 7]	[Donated 7 the last time (by the notes) and 6 this time.]; [Hesitant, but generous.]; [contributed 6]	[donated 10]; [Contributed 10 points]; [Gave all 10!]

	[Kept all but 2 points. Greedy.]	[Contributed 10 points]; [contributed all]	[They (and everyone else) gave 10. It was a good round!]	[Gave all 10.]; [Gave all, generous]
	[Donated 10 points]; [Gave the full 10. Clearly a mensch.]; [Contributed 10 points]	[donated full 10]; [Gave the full 10!]; [Donated everything. Good person.]	[They (and everyone else) gave 10. It was a good round!]; [Donated 10]	[0 contributed]; [0 points....]
**S7**	[Contributed 7]; [I gave 10 to the group, A gave 7]; [7 out of 10]	[5/10]; [Only contributed half and got biggest payout.]	[10/10. got mad heart!]; [10/10]	[shared 9]; [9]
	[only put in half, but still better than 0.]; [This person only contributed half of their amount and got the biggest payoff in the last round.]	[contributed 5]; [5/10]	[5 out of 10]; [5]	[6. One more than last round, according to note.]; [6/10]
	[This person contributed slightly more than half.]	[Gave 10]; [10]	[10]; [10/10]	[contrivuted 10]
	[put in a good 6 points, i think they will keep it up.]; [This person contributed slightly more than half.]	[8 out of 10]; [L gave 8, I plan to give 10]	[contributed 7]; [7/10]	[10]; [10]
		[They are very generous. Gave 10.]; [10/10]; [Contributed all 10 of their points.]	[10 out of 10]; [10]	[10/10. give this man a bonus!]; [contributed 10]
		[contributed 5]; [5/10]	[5]; [Only contributed half.]	[5]; [shared 5]; [5]
		[5/10. meh.]; [Gave half]; [5]	[shared 5]; [contributed 5]; [5/10]	[5 out of 10]; [5/10]; [5/10]
		[shared 5; [5 out of 10]; [P gave 5, I plan to give 10]	[5]; [5/10. meh...]; [5/10]	[5, same as last round]; [5/10]
	[I gave 10 to the group, B gave 6]; [6 out of 10]	[6/10]	[shared 10]; [10/10]	[10/10. another awesome player.]
	[Contributed 10]; [10 out of 10]	[7/10. yass.]; [7]	[Contributed a little more than half.]	[6/10]
	[Contributed 5]; [I gave 10 to the group, D gave 5]	[shared 10]; [D gave 10, I plan to give 10]	[10]	[3/10]; [3/10]

	[shared half]; [Contributed 5]	[They gave 9.]; [Contributed 9 of their points.]	[shared 7]; [contributed 7]	[5, less than last round]
	[Okay]; [shared half]; [Contributed 5]	[contributed full]	[8]; [8/10. OKAAAY...]	[10 out of 10]; [Contributed everything.]
	[Generous]; [Contributed 10]	[10/10. WINNER!]; [Gave 10]	[10 out of 10]	[10]; [shared 10]
	[Generous]; [shared all]	[shared 5]; [5 out of 10]	[7]; [Contributed 70% of their points.]	[0/10. what a loser.]; [contributed 0]
	[person I is very trustworthy, they put in all their pot!]; [This person is generous.]	[They gave 7.]; [7/10]	[5]	[3 out of 10]; [3/10]
**S15**	[Only contributed half]	[not bad]	[generous]; [contributed almost all points]	[Contributed all 10 points]; [Definitely a team player]
	[very generous]; [Donated the full 10 points]; [Very generous]	[Donated full amount]; [Contributed full amount]	[good contributor]; [Definitely a team player]	[greedy]
	[Contributed 10 out of 10.]; [Donated the full 10 points]; [Very generous]	[Definitely a team player]	[10/10 contributed]; [Contributed full amount]	[full]; [Donated full amount]
	[Contributed 8 out of 10.]; [slightly generous]; [generous]		[Donated full amount]; [gave 10]	[8/10]
		[7/10 contributed]	[10/10]; [Definitely a team player]	[very generous, contributed 10]; [Donated full amount]
	[Good job]	[Only donated 5/10]; [Donated half amount]; [Contributed half]	[only half]	[5/10]; [Contributed half of points (5)]
	[Good job]	[very greedy]	[Contributed half of points (5)]; [Donated half]; [gave 5]	[Contributed half]; [not a good contributor]
	[Good job]	[Contributed half of points.]	[5/5 contributed]; [greedy]; [Contributed half to group]	[5/10]; [greedy]
	[goes the distance]	[Team player, gave 10/10]; [Contributed full amount]	[Contributed 10 points]; [gave 10]	[very generous, contributed 10]; [full]
	[hope you have a great day]; [Contributed full	[generous]; [Definitely a team player]	[all]	[10/10]; [gave 10]

	amount]			
	[hope you have a great day]; [Contributed only 6]	[Contributed well at 9]	[9/10]; [good contributor]	[Contributed 8]; [team player]
	[Focused on the larger payoff for the group, contributed all monies]; [Very much a team player]	[10/10 contributed]; [Puts in a whole 10 for the group]; [contributed all]	[Contributed 10 points]; [Donated full amount]	[10/10]
	[Very much a team player]	[Team player, gave 10/10]; [Donated full amount]	[10/10 contributed]; [generous]	[team player]
	[allocated 7 to group, did well.]	[generous]	[9/10]; [good contributor]	[8/10]; [8/10]
	[allocated half to group]		[only half]	[very generous, contributed 10]; [full]; [Donated full amount]
	[Contributes next to nothing. 2 out of 10.]; [greedy]; [Donated only 2 points]	[5/10 contributed]; [They like to only put in 5]		[Contributed 8]
	[average contributor]	[Contributed less, but so did I, in Stage 1.]; [Stingy. Only contributed 2.]	[contributed only 1]; [Only contributed 1, stingy]	
		[Person J contributed 2, Person F contributed 10, and Person N contributed 3]	[contributed 5]	[Gave 10]; [generous contributor 10]
	[Nice contribution.]	[Gave half]; [average midway contributer]	[contributed 5]; [contributed only 5]	[Gave 5]; [contributed 5]
	[Good decision.]	[Not a good contributor. When everyone contributed 10, Person L contributed 5.]; [Contributed 5]; [put in 5, probly likes justin bieber]	[Gave half]; [Gave exactly half, 5]; [low contribution]	
	[Put in 5]	[same contribution as me]; [Fair contribution]	[contributed 5]	[Gave 4]; [contributed 4, eats babies]
	[Put in 2]	[Person J contributed 2, Person F contributed 10, and Person N contributed 3]	[below average contributor (3)]; [contributed 3.....probably punches babies in his free time]; [stingy.	

**S18**			Contributed least]	
		[a little above average contributer]	[Gave 7, better than half]; [decent contributor]	[Contributed 6]; [6]
	[Put in 10]	[Contributed 10]; [put in 10,]	[after getting notes that he contributed 10, only contributed 3 this time, will probably drop to 0 next time.]	[above average contributor 7]
	[great contributor]	[Person J contributed 2, Person F contributed 10, and Person B contributed 10]	[Gave 10]; [gave 10 like me]	[contributed 10, has a huge D.]
		[Gave 7]	[cont. 7, has a fairly large corgi.]	[Gave 10]
	[great contributor]	[Contributed 10 pts along with everyone else.]; [put in 10]		[Gave 8]; [8]; [gave a lot]
	[Person E contributed 2, Person G contributed 10, and Person F contributed 8]; [Greedy, probably listens to justin bieber.]	[Fair contribution]	[Gave 10]; [Gave the full 10]	[Gave 5]; [average contributor 5]
	[Average greed/selfless, names probably Tom.]		[contributed 4]	[Gave 0]; [contributed nothing]; [gave none!]
	[Person E contributed 2, Person G contributed 10, and Person F contributed 8]; [Selfless person, smells like bananas.]	[Gave all]; [very generous]	[Very generous, contributed 10]	
	[Person E contributed 2, Person G contributed 10, and Person F contributed 8]	[Contributed 10 pts along with everyone else.]; [Contributed 10]	[a little above average contributor (6)]	[Gave 5]
	[Nice job.]	[same contribution as me]	[average (5) contributer]; [contributed 5. average size.......... contribution.]	[Gave 5]; [contributed 5]; [5]

References

1. Hendriks, A. SoPHIE - Software platform for human interaction experiments. Working Paper (University of Osnabrueck, 2012).
2. West, B. T., Welch, K. B., & Galecki, A. T. (2014). *Linear Mixed Models: A Practical Guide Using Statistical Software*. (CRC Press, 2014).
